# Signalling Pinpointed to the Tip: The Complex Regulatory Network That Allows Pollen Tube Growth

**DOI:** 10.3390/plants9091098

**Published:** 2020-08-26

**Authors:** Patricia Scholz, Jannis Anstatt, Hannah Elisa Krawczyk, Till Ischebeck

**Affiliations:** Department of Plant Biochemistry, Albrecht-von-Haller-Institute for Plant Sciences and Goettingen Center for Molecular Biosciences (GZMB), University of Goettingen, Justus-von-Liebig Weg 11, D-37077 Goettingen, Germany; jannis.anstatt@stud.uni-goettingen.de (J.A.); hannah.krawczyk@uni-goettingen.de (H.E.K.)

**Keywords:** pollen tube, phosphoinositides, small G proteins, reactive oxygen species, phosphatidic acid, lipid signalling, cell wall, secretion, exocyst complex

## Abstract

Plants display a complex life cycle, alternating between haploid and diploid generations. During fertilisation, the haploid sperm cells are delivered to the female gametophyte by pollen tubes, specialised structures elongating by tip growth, which is based on an equilibrium between cell wall-reinforcing processes and turgor-driven expansion. One important factor of this equilibrium is the rate of pectin secretion mediated and regulated by factors including the exocyst complex and small G proteins. Critically important are also non-proteinaceous molecules comprising protons, calcium ions, reactive oxygen species (ROS), and signalling lipids. Among the latter, phosphatidylinositol 4,5-bisphosphate and the kinases involved in its formation have been assigned important functions. The negatively charged headgroup of this lipid serves as an interaction point at the apical plasma membrane for partners such as the exocyst complex, thereby polarising the cell and its secretion processes. Another important signalling lipid is phosphatidic acid (PA), that can either be formed by the combination of phospholipases C and diacylglycerol kinases or by phospholipases D. It further fine-tunes pollen tube growth, for example by regulating ROS formation. How the individual signalling cues are intertwined or how external guidance cues are integrated to facilitate directional growth remain open questions.

## 1. Introduction

Pollen are the male microgametophytes of seed plants, part of the short haploid phase in the life cycle of the spermatophytes. In angiosperms, the microgametophyte consists of one vegetative cell and two sperm cells formed from a generative cell, either during pollen development or later during fertilisation. After contact of the pollen grain with a compatible stigma, the vegetative cell forms a pollen tube that grows through the style to the ovule to transport the inherently non-motile sperm cells to the embryo sac [1,2]. There, the pollen tube ruptures and releases the sperm cells for double fertilisation, which concludes the gametophytic phase of the angiosperm life cycle [3,4,5].

Growth of the angiosperm pollen tube relies on extreme polarisation of the vegetative cell. Massive secretion of new cell wall material takes place at the very tip of the pollen tube, which exclusively extends into one direction. This extreme form of polarised cell expansion is called tip growth and occurs in plant pollen tubes as well as in plant root hairs [6,7]. The tip growth of pollen tubes reaches considerable growth rates in the range of 0.1 µm/s in vitro and 1 µm/s in vivo. Pollen tubes are thus among the fastest growing cells in the plant world [8]. The enhanced growth rate of angiosperm pollen tubes is thought to be a key factor contributing to the dominance of angiosperms in land plants today [9]. On the other hand, it poses significant challenges to cell organisation, cell wall integrity, secretion, and signalling networks regulating the complex process of tip growth.

## 2. Pectin Is an Important Component of the Pollen Tube Cell Wall

Pollen tube tip growth is based on a delicate equilibrium between secretion of cell wall material at the pollen tube tip and turgor-driven cell expansion. The maintenance of this equilibrium and concurrent preservation of the pollen tube’s shape sets special demand for the cell wall and its components. Modelling approaches demonstrated that a gradient in cell wall stiffness is required to sustain proper pollen tube shape [10,11,12,13]. This gradient changes from the more flexible apical tip towards the stiff pollen tube shank [13,14,15]. The cell wall component best suited to provide this gradient is pectin, a polymer characterised by its backbone containing galacturonan [12,16]. Pectin is synthesised in the Golgi apparatus and subsequently transported and secreted via secretory vesicles [17]. The apically secreted pectin is initially in an esterified state, as the carboxyl groups of the secreted galacturonan are linked to methoxy groups [16,18,19]. The esterified pectin is very flexible, allowing turgor-driven expansion. Upon cleavage of the ester bonds, pectin chains are cross-linked by Ca^2+^ ions, thus changing the rheological properties of the pectic compounds to increased stiffness [20,21]. Together with increasing abundance of cellulose and callose in the cell wall of the pollen tube shank, this leads to increased cell wall stiffness and stabilises the distal pollen tube cell wall. This results in a cell wall that resists the turgor and maintains the cylindrical shape of the pollen tube [14,22,23]. The transition from methylesterified “soft” pectin to de-esterified pectin occurs in the subapical region [14,19]. The enzymes responsible for this transition are pectin methylesterases (PMEs), that cleave the ester bonds in the secreted galacturonan monomers, enabling Ca^2+^-crosslinking of the pectin chains [24]. Lack of PME activity frequently leads to impaired pollen tube growth, visible in decreased growth rate and pollen tube length [20,25,26,27]. PMEs and pectin are transported in the same secretory vesicles and are subsequently secreted at the tip of the pollen tube. Consequently, the enzymatic activity has to be controlled to prevent premature de-esterification. According to protein structure type I and type II, PMEs are distinguished that also differ in their regulation mechanism [28,29]. In type II PMEs, premature enzyme activity and de-esterification of pectin is prevented by proteinaceous pectin methylesterase inhibitors (PMEIs) that form 1:1 complexes with the enzyme [30]. For type I PMEs, it is assumed that a protein-inherent pro-region acts as an auto-inhibitor during transport, as this pro-region shows significant homology to PMEIs [28,29,31]. The activation of type I PMEs requires the cleavage of the pro-region, and mature PMEs purified from the cell wall lack this domain [31]. However, where and how this cleavage occurs is still unclear. Activation of type II PMEs on the other hand can be achieved by the removal of PMEIs from the apoplast, which is attained by the endocytic uptake of the PMEIs in the subapical region of the pollen tube [32].

## 3. Pectin Secretion during Pollen Tube Growth

Providing enough pectin at the apical tip to maintain the fast growth rates of pollen tubes requires abundant secretion of cell wall material. Hence, exocytic vesicles fuse at a high rate with the pollen tube plasma membrane in the tip regions, providing signalling molecules as well as cell wall and membrane material [12,33,34,35]. Abundant endocytic processes balance exocytosis, which serves at least two functions: Firstly, to take up regulatory molecules from the apoplast, e.g., PMEI proteins, and secondly, to internalise surplus membrane material. Due to the higher surface-to-volume ratio of the delivered secretory vesicles, more membrane lipids than required fuse with the plasma membrane. These excess phospholipids are at least partially recycled back by the budding of endocytic vesicles [32,36,37,38]. The exact localisation of the exo- and endo-cytic processes is a matter of debate, that shall not be discussed here but has been examined elsewhere [6,39].

**Figure 1:**
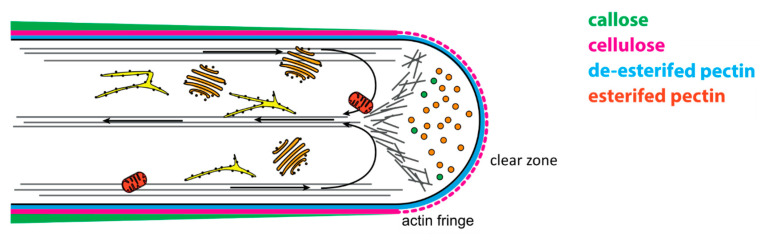
Tip-growing pollen tubes require a distinct subcellular organisation. The abundant secretion processes at the pollen tube tip lead to a distinct structuration in growing pollen tubes. The apex of the growing tube is packed with secretory vesicles and void of organelles leading to a V-shaped “clear zone”. It is speculated that the organelles are prevented from entering that zone by the cortical actin structure of the actin fringe. Behind the actin fringe, F-actin forms strong actin bundles required for transport processes. Vesicles and organelles, like mitochondria (depicted in red), endoplasmic reticulum (yellow), and Golgi (orange) structures, are transported on cortical actin bundles towards the tip. Retrograde transport takes place on central actin cables, resulting in a “reverse fountain” pattern of cellular transport. Additional structuration can be observed in the pollen tube cell wall. Esterified pectins predominantly form the cell wall at the pollen tube tip, although the presence of cellulose has been reported in some cases. Further back, cellulose and callose are embedded in a pectin matrix of de-esterified pectin monomers crosslinked with Ca2+ ions, leading to increased stiffness of the shank cell wall.

The precedence of endocytic and exocytic processes in the growing region of the pollen tube, aka the tip, is mirrored in the pollen tube’s subcellular structure (Figure 1). Growing angiosperm pollen tubes exhibit a pronounced zonation of the cytoplasm [33,40]. The apex of the growing tube is packed with secretory vesicles and lacks organelles, effecting a “clear zone” in light microscopy imaging [41,42,43]. The concentration of vesicles is maintained by F-actin structures, most visible in the form of the “actin fringe” in the subapical region adjacent to the clear zone [41]. This subapical region is organelle-rich and forms the transition between growing tip and non-growing shank-region of the pollen tube. The shank can be divided into the proximal and the distal end, the latter of which is highly vacuolated in older pollen tubes and separated by callose plugs from the actively streaming cytoplasmic shank region [34]. The proximal shank region is organelle-rich, containing, i.a., the male germ unit of the two sperm cells and the vegetative nucleus, which is transported on microtubules maintaining a constant distance to the pollen tube tip [44]. Additionally, endoplasmic reticulum (ER) and Golgi apparatus are present, which produce the contents of the secretory vesicles accumulating at the pollen tube tip. The movement of these organelles and their vesicles towards the apex strongly relies on actin structures. Throughout the shank, strong cortical and central actin bundles serve as tracks for associated myosins and their cargos. It is speculated that the actin fringe then serves as a filter that withholds the organelles in the subapical region and allows only vesicles to pass to the apex [8,41]. The transport of membranous organelles in the pollen tube of angiosperms follows a “reverse fountain” pattern: Organelles and exocytic vesicles are transported in anterograde direction to the tip close to the cell membrane, while retrograde transport occurs along central actin cables. The typical actin structure and the “reverse fountain” streaming pattern immanently lead to the V-shape of the clear zone at the apex, also termed inverted cone [42,44].

## 4. Pollen Tube Growth Is Regulated by Several Interwoven Signalling Networks

**Figure 2:**
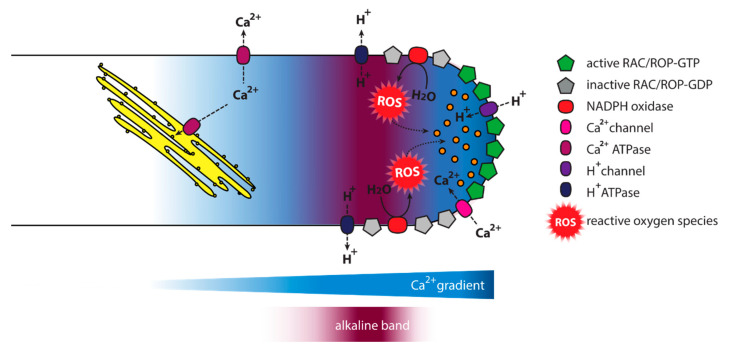
A complex signalling network is involved in pollen tube growth. Pollen tube growth is controlled by a variety of signalling factors, including ions, reactive oxygen species (ROS), and small Rop/Rac-GTPases cycling between active and inactive states. Furthermore, different phosphoinositides and derived lipids are involved in pollen tube growth regulation (See Figure 3 and Figure 4).

The equilibrium between the cell’s turgor and the secretion of pectin has to be accurately balanced to maintain proper tip growth [6], and the pollen tube must be able to redirect its growth direction in response to external guidance cues [45]. Consequently, a plethora of signalling networks and factors are involved in the regulation of pollen tube growth, including ion gradients, small GTPases, reactive oxygen species (ROS) (Figure 2), and signalling phospholipids [7,39,46,47,48,49,50,51]. A further layer of complexity is added by the cross-talk between the different signalling networks: GTPase activity influences the pollen tube inherent calcium gradient and relies on phosphoinositides for downstream signalling, while phosphoinositides influence the regulators of GTPases [52,53,54]. This leads in vitro to interdependent oscillations of GTPase activity, secretion, the Ca^2+^ gradient, and the growth rate observable, e.g., in lily or tobacco pollen tubes [55,56,57,58,59].

## 5. Reactive Oxygen Species in Pollen Tube Growth

ROS are vital signalling molecules during fertilisation, as they control stable pollen tube growth but also induce rupture of the tube at the end of its life cycle [51,60]. First indications on the role of ROS in pollen tube growth came from studies on the tobacco NADPH oxidase (NtNOX) [61]. NOX activity is confined to the plasma membrane and accumulation of ROS at the pollen tube tip could be visualised by dihydrofluorescein diacetate or nitroblue tetrazolium staining. Furthermore, inhibition of NOX activity disturbs pollen tube growth [61,62]. ROS production was also observed in lily, pear, kiwi, *Picea meyeri*, or olive pollen tubes [63,64,65,66,67]. In Arabidopsis, the two respiratory burst oxidase homolog (Rboh) genes, *AtRBOHH* and *AtRBOHJ*, are expressed in pollen tubes, where their encoded proteins localise to the apical plasma membrane. AtRBOHH and AtRBOHJ are enzymatically active in a Ca^2+^-dependent manner and pollen tube growth in the double mutant *rbohH rbohJ* is impaired [68]. Additional investigation of the *rbohH rbohJ* double mutant revealed higher amplitudes in growth rate oscillations and a tendency to burst prematurely. It was consequently proposed that AtRBOHH and AtRBOHJ serve as “speed control”, to allow coordination of cell expansion and deposition of new cell wall material [69]. This model was later extended, describing AtRBOHH and AtRBOHJ as part of a cell wall integrity-controlling pathway in pollen tube growth [50,70]. There, they are proposed as downstream effectors of a complex of receptor-like kinases (RLKs) [70,71,72], which responds to autocrine signalling peptides of the rapid alkalinization factor (RALF) family [4,35,71]. RALF4 and RALF19 act redundantly in the maintenance of pollen tube integrity as they influence the deposition of callose and pectins in the growing pollen tubes [35]. Pollen tubes of the double mutant *ralf4 ralf19* burst prematurely, likely due to their involvement in the secretion of cell wall material [4]. RALF4/19 interact with the *Catharanthus roseus* receptor-like kinase 1-like (CrRLK1L) receptors BUDDHA’S PAPER SEAL (AtBUPS) 1/2 and ANXUR (AtANX) 1/2, of which AtANX1/2 were shown to act upstream of AtRBOHH and AtRBOHJ [70]. Furthermore, addition of RALF4 led to increased ROS production in in vitro grown pollen tubes [71]. Another member of the RALF family, RALF 34, might play an antagonistic role to RALF4/19. RALF34 is predominantly expressed in mature ovules and its addition to pollen tubes grown in vitro induces pollen tube rupture [4]. Like RALF4/19, RALF34 is able to bind to ANX1/2 and BUPS1/2 and was proposed to outcompete RALF4/19 after the pollen tube reaches the ovule, thereby inducing pollen tube rupture [4,73]. Interestingly, pollen tube rupture and sperm release was also connected to high ROS concentrations in the filiform apparatus [60]; however, a direct connection between RALF signalling and ROS production by the female gametophyte has not been described so far.

Another described source of ROS in pollen tubes is oxidation of the polyamine spermidine catalysed by a polyamine oxidase [74,75]. Loss of polyamine oxidase activity leads to shorter pollen tubes and the tip localisation of its polyamine substrates seems to be important for physiological tip growth [75,76]. ROS signals are integrated into other signalling networks during pollen tube growth, including signalling phospholipids, small GTPases, and ion gradients [62,68,77,78]. For example, AtRBOHD triggered by abscisic acid (ABA) in stomatal closure has been shown to bind to the lipid phosphatidic acid, and phosphatidic acid increases ROS production [79]. Also, Ca^2+^ has been proposed to act both up- and down-stream of ROS signals, activating RBOH enzymes, whose ROS products could in turn trigger Ca^2+^-influx from the apoplast in a positive feedback loop [68,80].

## 6. Ion Gradients in Growing Pollen Tubes

Among ions, Ca^2+^ and H^+^ have been most closely linked to pollen tube growth regulation. In lily pollen tubes, a pH-zonation from the acidic tip towards an alkaline band at the base of the clear zone was described [81]. A similar pH gradient changing with growth rate oscillations was also observed in pollen tubes of tobacco and has been linked to the shank-localised, proton-exporting H^+^-ATPase NtAHA [82,83]. The role of H^+^ ions in the regulation of tube growth remains unclear though—initial studies on the pH gradient in *Lilium longiflorum* concluded that protons are not central to growth regulation [84]. However, later studies described a connection between pH and actin organisation during tube growth [85]. Additionally, reversible inhibition of pollen tube growth in lily using potassium cyanide (KCN) leads to a sharp decline of the pH gradient, and the alkaline band was the first ion gradient to be re-established after removal of the inhibitor [86]. A study on Arabidopsis pollen tubes connected intracellular pH changes with anion transport over the pollen tube membrane, hypothesising that protons might be part of a network reacting to γ-aminobutyric acid (GABA)-mediated signalling [87]. Further observations were made in a recent study on an Arabidopsis triple mutant lacking the three autoinhibited plasma membrane proton (H^+^) ATPase (AHA) isoforms, AtAHA6, AtAHA8, and AtAHA9 [88]. *AHA6*, *AHA8*, and *AHA9* are predominantly expressed in pollen and pollen tubes and pollen of the triple mutant *aha6 aha8 aha9* shows reduced germination rates, while germinated pollen tubes grow slower and tend to stop prematurely. AHA6, AHA8, and AHA9 localised to different extents in the plasma membrane of the pollen tube shank. There, they are thought to pump protons out of the pollen tube’s cytoplasm, thereby establishing a proton gradient from the tip towards the shank. Consequently, the cytoplasm of pollen tubes from the triple mutant *aha6 aha8 aha9* is more acidic and the pH gradient is strongly decreased. Further analysis of the mutant pollen tubes also revealed a decreased anion efflux from the pollen tube, a decreased membrane potential, and changes in the actin organisation, underlining the importance of proton transport across the pollen tube membrane [88].

The importance of Ca^2+^ for pollen germination and subsequent pollen tube growth has already been reported more than 50 years ago for more than 80 plant species [89]. Furthermore, the interaction between pollen tubes and the female gametophyte before double fertilisation is also accompanied by distinct Ca^2+^ dynamics in synergids, egg, and central cell [90,91]. In terms of pollen tube growth, it has been observed that growth rate oscillations and oscillations of the Ca^2+^ gradient depend on each other in vitro [92,93]. The Ca^2+^ gradient in the cytosol of growing pollen tubes is formed with peak concentrations close to the apical plasma membrane. The exact localisation of the peak Ca^2+^ concentration determines growth orientation [93,94]. Exact Ca^2+^ concentrations in the pollen tube vary considerably between reports, depending on the methods used [95]. At the tip, values are in the range of 0.1 to 10 µM, while for the shank, values around 0.02–0.2 µM have been reported [49,95,96]. Reports agree though, that the Ca^2+^ gradient is only observable in growing pollen tubes [95]. Irrespective of the actual values, the high apical concentration of Ca^2+^ is likely established by an influx of Ca^2+^ from the apoplast, as the application of calcium chelators and calcium channel inhibitors leads to the dissipation of the Ca^2+^ gradient [97,98]. Different Ca^2+^ channels at the tip of the pollen tube are one requirement and regulation point for a Ca^2+^ gradient [99,100,101,102]. In this context, Arabinogalactan proteins in the apoplast might play a role, acting in a feedback loop that couples Ca^2+^-influx and secretion with the physical strain at the pollen tube tip during cell wall extension [103]. Secondly, a mechanism for the efflux of Ca^2+^ in tip-neighbouring regions is required. This Ca^2+^ efflux occurs via PM- and possibly also ER-localised Ca^2+^-ATPases, transporting the ions out of the cell or into the endomembrane system, respectively [96,104,105].

Calcium could play a direct role in secretion by triggering exocytosis, as has been shown in neurons, where a calcium influx rapidly triggers the soluble *N*-ethylmaleimide-sensitive-factor attachment receptor (SNARE)-mediated vesicle fusion of neurotransmitter-filled vesicles with the plasma membrane. The transmitting of the Ca^2+^ signal is thought to occur via the two synaptic proteins Synaptotagmin-1 and Complexin, which interact with SNARE proteins on vesicles and the presynaptic membrane in a partially assembled state [106,107]. Interestingly, Soluble NSF attachment protein 25 (SNAP-25), a protein of the SNARE complex, was in turn reported to control voltage-gated Ca^2+^ channels and Ca^2+^ concentrations in the presynaptic cell [108]. While this process serves as an example that Ca^2+^ might directly influence secretion, it is unclear if Ca^2+^ in pollen tubes would act similarly, as secretory vesicles with cell wall material are unlikely to be in a similar “clamped” state of a partially assembled SNARE complex.

Furthermore, detection of Ca^2+^ occurs via sensor proteins, like calcium-dependent protein kinases (CDPKs). CDPKs possess a regulatory Ca^2+^-binding domain, and upon Ca^2+^-binding, the protein kinase catalytic domain is activated, so they act as “sensor responders” [49,109]. CDPK proteins involved in pollen tube growth have been reported for Arabidopsis, maize, and petunia [110,111,112], and CDPK activity has been linked to the adjustment of ion fluxes across the plasma membrane [113,114,115,116]. Other Ca^2+^ sensor proteins include calmodulin, calmodulin-like proteins, and calcineurin B-proteins that act as “sensor relays” transmitting the Ca^2+^ signal via altered protein–protein interactions. Several of these sensor relays have been described in connection with tube growth, actin organisation, and regulation of K^+^ transmembrane transport [117,118,119,120,121]. Ca^2+^-mediated stabilisation and destabilisation of actin filaments could also play a role in establishing actin structure, as high Ca^2+^ concentrations cause disassembly of filamentous actin, acting via actin-binding proteins [122]. This regulation of actin structure connects Ca^2+^ to another signalling factor as Ca^2+^-dependent F-actin modulation is one of two downstream pathways of the Arabidopsis Rop/Rac GTPase Rop1 [52].

## 7. Small GTPases Define the Pollen Tube Tip

The impact of small GTPases on tip-growing cells has been discussed in several recent reviews, therefore we will only briefly mention their basic roles and functions in pollen tube growth here [39,48,123,124]. Small monomeric GTPases (ca. 21 kDa) serve as molecular switches, cycling between an active GTP-bound state and an inactive GDP-bound state [125]. They are regulated by further proteins like guanine nucleotide exchange factors (GEFs), GTPase activating proteins (GAPs), and guanine nucleotide dissociation inhibitors (GDIs). GEFs activate small GTPases by exchanging GDP against GTP, while GAPs activate the intrinsically low GTPase activity of the monomeric GTPases [126,127]. GDIs modify subcellular localisation of small GTPases as they are able to remove the prenylated GTPases from the membrane and sequester them in the cytoplasm [128].

The superfamily of small GTPases is further divided into different GTPase families, i.a., the Rab- and the Rho-family [129]. Proteins of the Rab-GTPase family are involved in intracellular membrane trafficking during pollen tube growth, regulating vesicle transport between Golgi apparatus and ER or from the Golgi apparatus to the plasma membrane [130,131]. In Arabidopsis, the Rab-GTPase AtRabA4d is tip-localised and regulates vesicle trafficking, and its absence causes male-specific transmission defects [132]. Arabidopsis pollen tube growth is also impaired in null mutants of AtRabD2b and AtRab2c [133]. Importantly, Rab function relies on geranylgeranylation, and mutations of the Rab geranylgeranyl transferase inhibits normal pollen and pollen tube development, similar to mutations of the Rab proteins themselves [134]. In addition to the Rab-GTPase family, GTPases of the Rop/Rac protein family are involved in tip growth of pollen tubes. Active Rop-GTPases are locally confined to the pollen tube tip, thus providing a molecular marker for polarised secretion [54,135,136]. There, they can interact indirectly with the exocyst subunit Sec3, which determines polar secretion during pollen tube growth [137,138,139]. Furthermore, Rop-GTPases participate in the transduction of external signals like guidance cues, the organisation of the actin cytoskeleton, and the control of vesicle fusion with the tip plasma membrane [12,52,140,141,142,143]. Lee et al. described how the activation of two antagonistic downstream pathways via the ROP-interactive CRIB-containing (RIC) proteins RIC3 and RIC4 manipulate F-actin polymerization: RIC4-mediated stabilisation of F-actin cables promotes vesicle transfer towards the tip [141]. However, RIC4-induced changes of actin structure impaired vesicle fusion at the pollen tip, which could be balanced by RIC3- and Ca^2+^-mediated F-actin disassembly. Thus, the authors suggested that Rop-GTPase activity triggers actin polymerisation for vesicle transport to the tip via the RIC4 pathway and actin depolymerisation at the tip for vesicle fusion via the RIC3 pathway. Together, these two pathways enable the transport and secretion of cell wall material at the tip [141]. Rop-controlled cell wall deposition relying on the manipulation of F-actin structure has also been observed in other context, e.g., the shaping of xylem vessels [144].

Studies in Arabidopsis characterised AtRop1 (also named Rac11 and Arac11) as a regulator of polarised pollen tube growth whose geranylgeranyl-mediated but spatially confined localisation to the tip is required for cell polarisation [145]. A second Arabidopsis enzyme, AtRop5 (also named Rac2, Rac6, and Arac6), seems to act similarly, and induces severe depolarisation phenotypes upon transient expression in tobacco [54]. Due to its close phylogenetic relationship, AtRop3 might have a similar function in pollen tubes [146]. In tobacco, NtRac5 has been described to regulate pollen tube growth in interaction with NtRhoGAP1 and NtRhoGDI2 [147,148]. AtRop1 was also proposed to be a mediator for changes in growth direction in response to pollen tube guidance cues. In a model that describes pollen tube tip growth as a result of the exocytosis-mediated polarisation of Rop1 and the secretory cell wall extension, guidance cues were predicted to shift the localisation of Rop1 activity and thus change growth directionality [12]. In this model, Rop1 activity is proposedly modulated by the activities of RopGEFs and the RhoGAP ROP1 ENHANCER 1. RopGEFs were then proposed as integration points for guidance cues, as an increased activation of Rop1 in response to a guidance cue would cause the shift in Rop1 activity and re-direction of secretion [12]. Consequently, RopGEFs, RhoGAPs, and RhoGDIs are important modulators of pollen tube growth due to their regulation of Rop/Rac GTPases [124,136,148,149,150]. Thus, male transmission defects were observed when RhoGDI function was missing in Arabidopsis [149]. Additional layers of modulation are characterised, e.g., phosphorylation of RopGEFs by cytoplasmic or receptor-like kinases, which was also described as a link between external signals and change of pollen tube growth directionality [143,151,152]. Guidance cues by the female gametophyte are required for precise pollen tube growth towards the micropyle [153]. Among the guidance cues described in Arabidopsis are the cysteine-rich AtLURE1-peptides, which are secreted from the synergid cells [154]. Reception of the AtLURE1 signals at the pollen tube relies on pollen-specific receptor-like kinases (PRKs) [143]. Specifically, AtPRK6 was determined as one receptor of AtLURE1 peptides as semi-in vivo grown pollen tubes of *prk6* mutants did not react to recombinant AtLURE1.2. AtPRK6 displayed plasma membrane localisation at the pollen tube tip, and interestingly, an asymmetric accumulation of AtPRK6 towards externally applied AtLURE1 was observed, preceding the change of pollen tube growth direction. Signal transduction of AtPRK6 requires its interaction with pollen RopGEFs via a cytosolic domain: complementation of the AtLURE1-insensitivity in *prk6* was not achieved when PRK6′s cytosolic interaction domain to RopGEFs was missing [143]. Other receptor-like kinases present in Arabidopsis pollen tubes were also shown to react to AtLURE1 peptides [155]. The ectodomains of recombinant MALE DISCOVERER1 (AtMDIS1) and MDIS1-INTERACTING RECEPTOR-LIKE KINASE (AtMIK) 1/2 bind to AtLURE1.2. AtMDIS1 can directly interact with both AtMIK proteins and this interaction is enhanced by AtLURE1.2. However, unlike for AtPRK6, no connection to RopGEFs has been described, with signal transduction likely relying on transphosphorylation in the AtMDIS1–AtMIK complex [155]. It thus remains to be uncovered to which extent RhoGTPase activity is involved in pollen tube guidance and which role is played by other signalling modules.

Lipid signals act together with the mentioned regulatory proteins in the regulation of Rop GTPases [53,54]. Phosphatidylinositol 4,5-bisphosphate (PI(4,5)P_2_) acts upstream of Rop GTPase signalling by promoting plasma membrane localisation of GTP-Rop, which is part of a complex feedback loop to maintain the tip localisation of active Rop [53,136]. Due to the diverse membrane trafficking processes, the active enzyme constantly shifts to the flanks of the pollen tube tip. There, it has to be inactivated by laterally localised RhoGAP proteins [135]. RhoGDIs remove the inactive Rop GTPase from the subapical membrane and the RhoGDI/Rop-heterodimer is then transported in the cytoplasm back to the tip [147]. Tip-localised PI(4,5)P_2_ shifts the Rop GTPase equilibrium between cytoplasm and membrane towards a membranous localisation, and RopGEFs complete the cycling of the enzyme by exchanging GDP against GTP, activating Rop GTPase [53,136,156]. However, PI(4,5)P_2_ was also described to act downstream of Rop GTPases. Tip-localised active AtRop5 interacts with a phosphatidylinositol 4-phosphate 5-kinase (PI4P5K), affecting the production of PI(4,5)P_2_, which controls subsequent downstream pathways targeting the actin cytoskeleton and vesicle fusion [54].

## 8. Phosphoinositides and Derived Lipids form a Signalling Network

**Figure 3:**
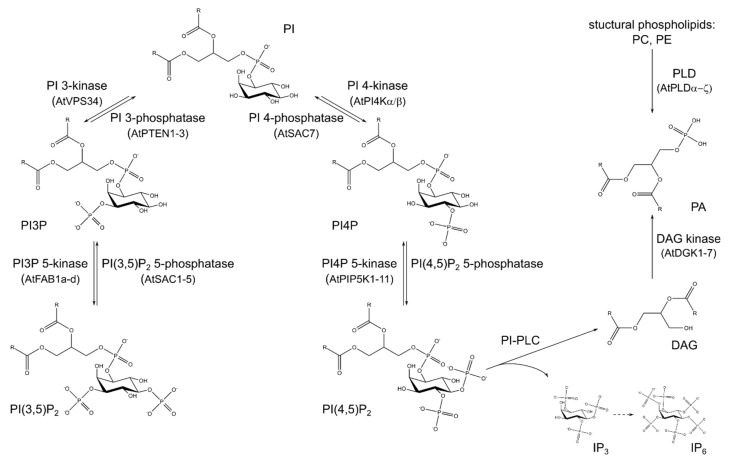
Signalling lipids are interconverted by a variety of enzymes. Phosphoinositides are phosphoglycerolipids carrying an inositol headgroup. Phosphorylation of different hydroxyl groups of the inositol in phosphatidylinositol (PI) gives rise to different mono- and diphosphorylated phosphoinositides. Phospholipase C-activity cleaves the bond to the *sn3*-hydroxyl group of the glycerol backbone, yielding diacylglycerol (DAG) in the process, which can be phosphorylated to phosphatidic acid (PA). Used abbreviations: DAG—diacylglycerol, IP_3_—inositol 1,4,5-trisphosphate, IP_6_—inositolhexakisphosphate, PA—phosphatidic acid, PI—phosphatidylinositol, PI3P—phosphatidylinositol 3-phosphate, PI4P—phosphatidylinositol 4-phosphate, PI(3,5)P_2_—phosphatidylinositol 3,5-bisphosphate, PI(4,5)P_2_—phosphatidylinositol 4,5-bisphosphate, PI-PLC—phosphoinositide phospholipase C, PLD—phospholipase D.

As becomes evident from the dependency of GTPase signalling on downstream signals like PI(4,5)P_2_, the phosphoinositide signalling network (Figure 3), derived phospholipids, and the respective enzymes are crucial for normal pollen development and tube growth. The phosphatidylinositol headgroup of phosphoinositides protrudes from the plasma membrane, thus providing an anchor point for proteins with respective phosphoinositide-binding domains [7,157]. These binding domains can also be used for visualising the different lipids, as specific domains bind to the differently phosphorylated subspecies of phosphoinositides [158].

## 9. All Phosphoinositides Derive from Phosphatidylinositol

All phosphoinositides derive from phosphatidylinositol (PI) as a biochemical precursor, which is synthesised in the membrane of the ER by phosphatidylinositol synthases (PISs) from the precursors CDP-diacylglycerol (CDP-DAG) and D-*myo*-inositol (Figure 3) [159,160]. Two PIS enzymes, AtPIS1 and AtPIS2, are described in Arabidopsis [159,160]. Interestingly, the metabolic fate of PI derived from AtPIS1 or AtPIS2 seems to differ. In Arabidopsis plants overexpressing *AtPIS2*, higher levels of phosphoinositides were detected in contrast to overexpression of *AtPIS1* that caused an increase of structural phospholipids [160]. Transient overexpression of *AtPIS1* or *AtPIS2* in tobacco pollen tubes led to wavy growth phenotypes [161]. In line with the proposed different metabolic fates of PI produced by AtPIS1 or AtPIS2, expression of *AtPIS2* led to a higher amount of affected pollen tubes. Similarly, co-expression effects of *AtPIS2* with other genes encoding for enzymes of the phosphoinositide network were far more pronounced than for *AtPIS1* [161].

The inositol of PI can carry phosphate groups in different positions, the D3-, D4-, or D5-position of the polyalcohol, giving rise to a number of different phosphoinositides. In plants, phosphatidylinositol 3-phosphate (PI3P), phosphatidylinositol 3,5-bisphosphate (PI(3,5)P_2_), phosphatidylinositol 4-phosphate (PI4P), and phosphatidylinositol 4,5-bisphosphate (PI(4,5)P_2_) have been characterised in different cellular functions. Of those, PI4P and PI(4,5)P_2_ are especially important in secretory processes and pollen tube growth [7,162,163], as will be outlined in the next sections.

## 10. PI4P Has Regulatory Roles in the *Trans*-Golgi Network

**Figure 4:**
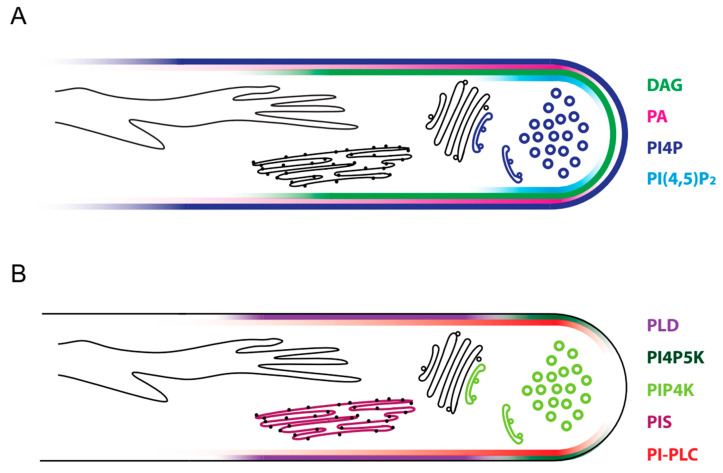
Several signalling lipids and the enzymes involved in their synthesis and degradation are localized at the apical plasma membrane. Several phosphoinositides and derived lipids are involved in pollen tube growth regulation. Their specific localisations in the plasma membrane are positional markers in secretory and endocytic processes and can be detected with the help of fluorescent lipid sensors (**A**). Enzymes acting on lipids of the phosphoinositide network show overlapping localisation to their products (**B**).

Phosphatidylinositol 4-phosphate (PI4P) is the major monophosphorylated phosphoinositide [164,165]. Localisation studies in Arabidopsis demonstrated the presence of PI4P in the plasma membrane, recycling endosomes, early endosomes, the *trans*-Golgi network, and the Golgi complex [166]. Estimating from fluorescence intensity and affinity of the used lipid sensors, PI4P abundance seems to follow a gradient, with the highest PI4P amounts in the plasma membrane and lowest amounts in the Golgi complex [166]. In tip-growing root hairs, PI4P concentrations were highest in the plasma membrane following a tip-focused gradient [167,168]. A similar membrane localisation was observed in growing tobacco pollen tubes overexpressing a fluorescent PI4P-reporter [161] (Figure 4A).

Conversion of PI to PI4P is catalysed by phosphatidylinositol 4-kinases (PI4Ks). Among these, PI4Kβ1 and PI4Kβ2 are especially important for tip growth. In the Arabidopsis double mutant *pi4kβ1 pi4kβ2*, pollen tubes exhibit a wavy growth pattern and are shorter in length; furthermore, root hairs are also shorter and display aberrant growth morphologies. PI4Kβ1 overexpression in tobacco pollen tubes stimulates pectin secretion [161]. For AtPI4Kβ1, physical protein interaction with AtRab4Ab and AtRab4Ad was demonstrated and electron tomography imaging of Arabidopsis root cells showed that both AtRab4Ab and AtPI4Kβ1 localise to the *trans*-Golgi network (TGN) [132,169,170]. Electron tomography images of root cells further indicated that PI4Kβ1 and PI4Kβ2 might be vital for size determination of secretory vesicles at the TGN [169]. However, as the TGN integrates exocytic and endocytic vesicles, it cannot be determined if defects of PI4Kβ impair exocytic or endocytic processes. In accordance with the described TGN-localisation, transient overexpression of *AtPI4Kβ1* in tobacco pollen tubes causes alterations of the TGN morphology. In addition, in the Arabidopsis mutant *lot*, which displays defects in TGN formation in pollen grain and pollen tubes, normal PI4P membrane localisation is abolished and *lot* plants are male-sterile [161,171]. Further studies showed additional signalling functions of PI4P and AtPI4Ks in vesicle trafficking and TGN organisation during cytokinesis and lateral root formation [172,173]. Although its synthesis is particularly linked to the TGN, PI4P strongly localises to the plasma membrane at the pollen tube tip. This discrepancy raises the question of how PI4P reaches the plasma membrane. One way could be via secretion of PI4P-loaded vesicles derived from the TGN. It is however also possible that PI4P is directly synthesized at the plasma membrane. Enzymes that might be responsible are PI kinase isoforms of the α subfamily that are predicted, for example, in Arabidopsis based on sequence homology [174]. Interestingly, in yeast, two types of PI4-kinases exist, one of which localizes to the plasma membrane [175,176].

At the plasma membrane, PI4P could have a function in its own right, but can also act as a substrate for phosphatidylinositol 4-phosphate 5-kinases (PI4P5Ks) that are localised in the same plasma membrane sub-domain [7,161].

## 11. PI4P 5-Kinases Catalyse the Formation of PI(4,5)P_2_ at the Pollen Tube Tip

PI4P5Ks catalyse the phosphorylation of PI4P to PI(4,5)P_2_. Arabidopsis PI4P5Ks come in two types: AtPI4P5K10 and AtPI4P5K11 belong to type A, while AtPI4P5K1 and AtPI4P5K9 belong to type B [174]. Both types contain a C-terminal catalytic and a dimerisation domain, however, type B AtPI4P5Ks possess an additional N-terminal Membrane Occupation and Recognition Nexus (MORN)-domain unique to plants, which is involved in regulation of enzyme activity [174,177] and a linker region necessary for correct subcellular localisation [178].

According to transcriptomic studies, several Arabidopsis *PI4P5Ks* are expressed to varying degrees in the different stages of pollen development and during pollen tube growth [179,180]. For *AtPI4P5K4*, *AtPI4P5K5*, *AtPI4P5K10*, and *AtPI4P5K11*, expression in pollen and pollen tubes of Arabidopsis was also verified histochemically, using promoter-β-glucuronidase-fusion [53,181]. Furthermore, proteomic analyses detected AtPI4P5K4, AtPI4P5K5, and AtPI4P5K6 in mature pollen grains [182]. Transient expression of the respective gene in tobacco pollen tubes revealed a plasma membrane localisation of the PI4P5Ks at the subapical flanks during pollen tube growth (Figure 4B). After cessation of growth, PI4P5K localisation expanded to the extreme apex [53,181,183,184]. In parallel, PI(4,5)P_2_ localisation has been studied using fluorescent lipid sensors similar to the previously described phosphoinositides. In accordance with the plasma membrane localisation of its biosynthetic enzymes, PI(4,5)P_2_ is detected almost exclusively at the plasma membrane of pollen tubes but also other cells, e.g., in the root cortex [53,54,166,181]. Furthermore, plasma membrane localisation in growing pollen tube membranes is not uniform, instead, PI(4,5)P_2_ is present in a distinct subapical domain that excludes the very tip (Figure 4A). Apical localisation of PI(4,5)P_2_ can only be found in pollen tubes that ceded to grow [53,181,185].

Considering the tip-localisation of both PI4P5Ks and PI(4,5)P_2_, it is not surprising that strong tip growth phenotypes are observed for several AtPI4P5K enzymes. Overexpression of type A PI4P5Ks, *AtPI4P5K10* and *AtPI4P5K11*, induces severe tip swelling and disturbance of the actin structure in tobacco pollen tubes, the overproduced PI(4,5)P_2_ inhibits RhoGDI and thus leads to activation and depolarisation of NtRac5 [53]. For *AtPI4P5K4*, *AtPI4P5K5*, and *AtPI4P5K6*, overexpression phenotypes with excessive pectin accumulation are observed. The affected pollen tubes are often branched or stunted in growth, with the protoplast “trapped” behind the accumulated pectin at the tip [181,183,184]. Enzyme activity of PI4P5K6s from Arabidopsis and tobacco is further regulated by mitogen-activated protein (MAP) kinase (MPK)-mediated phosphorylation. MPK activity seems to diminish PI(4,5)P_2_-production, and when *MPK* was overexpressed with *PI4P5Ks* in tobacco pollen tubes, it could reduce the aberrant pollen tube growth phenotypes [186]. The PI4P5K-produced PI(4,5)P_2_-signal is processed, i.e., via PI(4,5)P_2_-interacting proteins, regulating endo- and exocytosis. The Arabidopsis clathrin assembly protein EPSIN-LIKE CLATHRIN ADAPTOR PROTEIN 2 (ECA2/ PICALM5a) interacts with PI(4,5)P_2_ in vitro and pollen tubes of the *eca2* T-DNA insertion Arabidopsis line grow shorter [187].

PI4P 5-kinases also play a role in polar tip growth and secretory processes of non-plant species [188,189,190]. In the nervous system, for example, PI4P is required for the normal function of several ion channels including calcium channels important for secretion [191]. However numerous other interaction partners are known, such as SNARE proteins and the proteins CAPS and Munc13 that prime vesicles for exocytosis [192]. It will be interesting to find out how conserved the role of PI(4,5)P_2_ in secretion is across Eukaryotes. One complex that has already been shown to bind PI(4,5)P_2_ across kingdoms [192,193,194] is the exocyst complex, that is crucial for secretory processes.

## 12. PI(4,5)P_2_ Interacts with Components of the Exocyst Complex

Targeting of secretory vesicles to the correct destination is determined by tethering complexes that physically connect vesicles to the target membrane. Among them, the exocyst complex is especially important for polarised exocytosis by tethering post-Golgi vesicles to the plasma membrane. It localises to sites of maximal exocytosis in a variety of model organisms, relying on interactions with both phospholipids and small monomeric G-proteins [195,196,197]. The complex was originally discovered in yeast but later found to be conserved in all eukaryotes, including plants [198,199,200]. The exocyst complex consists of eight subunits, termed SEC3, SEC5, SEC6, SEC8, SEC10, SEC15, EXO70, and EXO84. SEC5 is the core subunit and connects many other subunits with each other, SEC15 binds to vesicles, SEC6 interacts with SNARE proteins, and both SEC3 and EXO70 bind to PI(4,5)P_2_ on the target membrane [137,195,197,199,201,202,203]. However, unlike yeast, which has only one homologue of each subunit, Arabidopsis possesses two homologues of *SEC3*, *SEC5*, and *SEC15* each, three homologues of *EXO84* and, strikingly, 23 homologues of *EXO70* in its genome [198,204].

First connections between the exocyst complex and PI(4,5)P_2_ signalling were observed in yeast. There, the SEC3 homologue Sec3p contains a pleckstrin homology (PH) domain in its N-terminal region, which interacts with PI(4,5)P_2_, mediating membrane binding [196,197,205]. The G-protein Rho1p can interact with the PH domain, presumably only if it is already bound to PI(4,5)P_2_ [203]. Yeast Exo70p directly interacts with PI(4,5)P_2_ through a polybasic region in its C-terminal domain and membrane binding of Exo70p was predicted to induce the local clustering of PI(4,5)P_2_, potentially inducing formation of exocytic hotspots [203]. Mutation of the residues responsible for PI(4,5)P_2_-binding leads to loss of Exo70p from the membrane, but not to secretion defects, while a combination of Exo70p and Sec3p mutations that leaves both proteins unable to bind lipids, was shown to be lethal in yeast [195,197].

Pollen tubes of Arabidopsis *sec3a*, *sec5*, *sec6*, *sec8*, *sec15a*, and *exo70C2* mutants showed male-specific transmission defects as germination and pollen tube growth were disrupted [137,199,206,207]. For the two Arabidopsis SEC3 proteins, some homology to yeast Sec3p could be observed: the N-terminal PI(4,5)P_2_-binding PH domain is conserved, however the Rho-interaction motif is not [137,138]. The PH domain of AtSEC3A is necessary and sufficient for membrane targeting during heterologous expression in tobacco pollen tubes but dispensable during homologous expression in Arabidopsis pollen tubes [137]. Similarly, PI(4,5)P_2_ is not required for the localisation of AtSEC3A to the germination site in Arabidopsis pollen [208]. Both studies indicate that AtSEC3A can localise to the membrane independently of PI(4,5)P_2_, possibly relying on other exocyst components or ROP G-proteins. Indeed, the Arabidopsis adapter protein ROP Interactive Partner 1 (ICR1) was found to compensate for the missing Rho-interaction motif of AtSEC3A by connecting it to the G-protein AtROP10 [138]. Whether similar adapter proteins play a role in pollen tube growth is not yet known.

Analysis of possible functional homology of the second yeast exocyst protein with PI(4,5)P_2_-binding ability, Exo70p, is impeded by the massive expansion of EXO70 in plants. The reason for this massive expansion is so far unclear, although different explanations have been proposed, e.g., tissue-specific expression, differences according to target sites, or specialised roles in cellular processes like autophagy, cytokinesis, or pathogen infection [209,210,211,212,213]. The different EXO70 isoforms might also be capable of binding different lipids [209]. Indeed, in mature trichomes, AtEXO70H4 is selectively targeted to the PA/PS-rich apical membrane domain, whereas AtEXO70A1 is selectively targeted to the PI(4,5)P_2_-rich basal membrane domain [214]. In tobacco pollen tubes, NtEXO70A1a and NtEXO70B1 each localise to distinct parts of the plasma membrane, which both partially co-localise with PA and PI(4,5)P_2_ [215].

Independent of lipid binding capabilities, different EXO70 family members also might have different functions in pollen tube growth. While there are discrepancies in the described gene expression, most reports agree on the expression of *AtEXO70A2*, *AtEXO70C1*, *AtEXO70C2*, *AtEXO70H3*, and *AtEXO70H5* in pollen tubes [204,207,215,216]. In most tissues of Arabidopsis, the prevalent EXO70 isoform that mediates polar exocytosis is AtEXO70A1. Although germination of *exo70A1* mutant pollen is impaired, pollen tubes appear to grow normal, so its functions are probably complemented by AtEXO70A2 [204,207]. AtEXO70C1 and AtEXO70C2 likely serve functions distinct from the main exocyst complex, since they do not interact with the core exocyst subunits. However, AtEXO70C1 and AtEXO70C2 interact with AtROH1, a member of the DUF793 protein family, which acts as a negative regulator of exocytosis [207]. Roles of AtEXO70H3 and AtEXO70H5 in pollen tubes are so far undescribed, however, other plant EXO70H proteins have been described to localise to the nucleus with possible functions independent of the core exocyst complex [209,215,217]. The tobacco NtEXO70B1 is expressed in pollen tubes and the encoded protein localises to the shank region of growing pollen tubes, partially co-localising with NtSEC3 and the site of endocytosis. This suggests that it might function within the exocyst complex in the coordination of endocytosis [215].

In agreement with its function in yeast exocytosis, the exocyst in plant pollen tubes is widely believed to guide exocytosis during tube growth. Different exocyst subunits localise to the described sites of exocytosis in the apex and/or the subapical zone, including SEC3, SEC6, SEC8, and EXO70A [137,199,215]. Additionally, slight shifts in the localisation of AtSEC3a and AtSEC8 were shown to precede changes in the direction of pollen tube growth [137]. Nonetheless, the molecular mechanism of the exocyst complex in pollen tube growth is still unclear. Early reports in budding yeast suggested that the lipid binding Sec3p and Exo70p might bind to the membrane, marking hotspots of exocytosis, while the other exocyst subunits bind to post-Golgi vesicles. Formation of the complete exocyst complex would then serve to tether vesicles to their correct target membrane [195,218]. In Arabidopsis root epidermal cells, however, it was reported that none of the exocyst subunits depend on vesicular traffic to localise to the membrane, indicating that the exocyst complex could pre-form on the target membrane and only then bind to vesicles [219]. On the other hand, AtSEC3a was shown to decorate vesicles in the inverted cone region of pollen tubes [137].

Taken together, molecular mechanisms of the exocyst complex in pollen tube growth remain elusive. Additional research will be required to fully resolve the order and the mechanism of interaction between exocyst, vesicles, and PI(4,5)P_2_ in the target membrane and the connections of this complex to the signalling networks important for pollen tube growth.

## 13. Dephosphorylation Reactions on PI4P and PI(4,5)P_2_

The strong phenotypes resulting from the overexpression of PI 4-kinases and PI4P 5-kinases illustrate that the levels of PI4P and PI(4,5)P_2_ must be tightly controlled. Furthermore, it seems crucial that the subdomain of the plasma membrane decorated with phosphoinositides remains clearly confined requiring the degradation of phosphoinositides diffusing out of this area. Therefore, phosphoinositides need to be degraded either by cleaving off their headgroup or by dephosphorylation. A cleavage reaction degrades the phosphoinositide “for good”, yielding a lipid and a polyphosphorylated inositol as reaction products. In contrast, a dephosphorylation reaction takes a phosphoinositide one step back in the phosphorylation cascade so that it can be quickly rephosphorylated. Due to this, the dephosphorylation reactions of PI4P and PI(4,5)P_2_ have potential for a fine-tuning of PI4P and PI(4,5)P_2_ concentrations. However, data on respective enzyme function in pollen tubes is scarce. The phosphatase ROOT HAIR DEFECTIVE4 (RHD4/SAC7) acts on the D4-phosphate in PI4P and is required for physiological tip growth in root hairs [7,167]. The protein has been detected in mature pollen grains and transient co-expression in tobacco pollen tubes showed a partial overlap in localisation with AtRabA4d in the endosomal compartment [132,182]. AtSAC7 is part of clade II of the suppressor of actin (SAC) phosphatases together with AtSAC6 and AtSAC8 [220]. Hence, it seems likely that AtSAC6 and AtSAC8 are PI4P-phosphatases as well, especially since all three are able to complement the yeast *sac1* null mutant in a similar manner, yet there are no reports on the enzymatic activity of AtSAC6 and AtSAC8 [221]. Expression of *AtSAC6* has been reported to be pollen-exclusive, especially high during pollen maturation, and it was found in a mutant screen to be required for β-aminobutyric acid-induced sterility [221,222]. A SAC protein homologue in rice acting on PI4P and PI(4,5)P2 was linked to actin polymerisation and adjustment of cell elongation [223]. However, despite their described involvement in cell growth and tip growth in root hairs and their expression in pollen, no connection to pollen tubes has been described so far for the clade II SAC phosphatases.

The clade III protein of Arabidopsis SAC phosphatases, AtSAC9, has been proposed to act on PI(4,5)P_2_ and inositol 1,4,5-trisphosphate and in roots of the respective mutant these two compounds accumulated [224]. The *sac9* mutant further showed a constitutively stressed phenotype, which was later connected to defects in cell wall structure and disorganised deposition of cell wall material [224,225]. Other phosphatases acting on the D5-phosphate in PI(4,5)P_2_ have been found in the protein family of plant 5′ phosphatases (5PTases) [226]. Arabidopsis possesses 15 proteins of the 5PTase family, which can be divided into two different groups based on their protein structure. At5PTase1-11 form group I, while group II contains At5Ptase12-15 (At5PTase15, mainly labelled AtFRA3) [226,227]. Substrate specificity is mixed in those groups, as different members from both groups show in vitro substrate preferences towards soluble inositol polyphosphates or hydrophobic phosphoinositides including PI(4,5)P_2_ [226]. From group I At5PTases, only *At5PTase5* is consistently expressed during different stages of the pollen life cycle, the corresponding protein was not found in mature pollen though [179,180,182]. In addition, involvement of the respective protein, variously labelled BST1, DER4, or MRH3, in the actual tip growth processes of root hairs or pollen in Arabidopsis seems to be minor [228,229]. The group II At5PTases genes *At5PTase12–14* are expressed during pollen development and tube growth according to transcriptomic data, and all three encoded proteins are detected in mature pollen [179,180,182]. These three proteins have been characterised enzymatically, which revealed in vitro substrate preference for PI(4,5)P_2_ in case of At5PTase14 and inositol 1,4,5-trisphosphate in case of At5PTase12/13. However, their in vivo substrate is unclear [230]. Hence, their role in the phosphoinositide network and possible impact on pollen tube growth and regulation of cell wall secretion remains to be studied.

## 14. Activity of Phospholipase C Is the Main Degradation Route of Phosphoinositides

In addition to modifications of their headgroup, phospholipids can be metabolised by phospholipases like phospholipase C (PLC) and phospholipase D (PLD) [231,232,233]. Cleavage of phospholipids by phospholipases C (PLCs) yields diacylglycerol (DAG) and the phosphorylated headgroup as products [232,233]. PLCs acting on lipid substrates can be divided into non-specific phospholipases C (NPCs) and phosphoinositide-phospholipases C (PI-PLC). PI-PLCs specifically use phosphoinositides as a substrate, while NPCs act on structural phospholipids like phosphatidylcholine (PC) or phosphatidylethanolamine (PE) [234,235]. Influences of Arabidopsis NPCs on root growth and pollen development but not pollen tube growth have been described [236,237,238]. In addition, so far, no Arabidopsis PI-PLC mutant was reported to be impaired in pollen tube growth. However, studies in other model systems highlighted PI-PLC importance to counterbalance PI4P5K-activity [185,239,240]. Petunia PiPLC1 and tobacco NtPLC3 both localise to the plasma membrane in growing pollen tubes, in overlapping regions with their substrate PI(4,5)P_2_, although the protein localisation tends to elongate further towards the shank [185,239]. A similar co-localisation was observed for NtPLC3 and the Arabidopsis PI4P5Ks AtPI4P5K2 and 11 after transient expression in tobacco pollen tubes [240]. For petunia PiPLC1, image time series indicated that the PLC localisation extends towards the extreme apex in phases of reduced growth and is less apical in phases of rapid growth [185]. Dominant negative expression of PLCs mutated in their active site or chemical inhibition of NtPLC3 caused a depolarisation of tip growth and swollen tube tips [185,239,240]. Additionally, PI(4,5)P_2_ visualised by a lipid sensor was observed to spread further towards the shank upon PLC-inhibition [239]. The pollen tube tip phenotype of swollen tips in tobacco pollen tubes was observed upon overexpression of inactive NtPLC3 and AtPI4P5K2; however, AtPI4P5K2-induced tip swelling could be reversed by simultaneous overexpression of active NtPLC3 [240]. Taken together, PLCs seem to be important in pollen tube tip growth as the main antagonist of PI4P5Ks, regulating PI(4,5)P_2_ abundance and location.

## 15. Inositol(poly)phosphates Are Receptors’ Cofactors

On the one hand, PLC activity abolishes the PI(4,5)P_2_-signal, while on the other hand, it produces two new molecules, inositol 1,4,5-trisphosphate (IP_3_) and DAG. Cleavage of PI(4,5)P_2_ to the second messengers IP_3_ and DAG is a common signal pathway in animal cells: DAG subsequently activates protein kinase C (PKC) while an IP_3_-receptor triggers the release of Ca^2+^ from intracellular storage [241]. However, neither for PKC nor for a canonical IP_3_-receptor do homologues exist in the Arabidopsis genome, so PLC-dependent responses seem to be transmitted differently [242,243]. In case of IP_3_, it was proposed that it rather acts as a precursor for higher phosphorylated inositol phosphates (IPs) and inositol pyrophosphates (IPPs) species like IP_5_, IP_6_, IP_7_, or IP_8_ [242,244,245]. A signalling role for IPs was, for example, shown in the hormone signalling pathways of auxin and jasmonates, as the TIR1- and COI1-receptor need the cofactors IP_6_ and IP_5_, respectively [246,247,248,249,250]. Both methyl jasmonate [251] and the auxin indole acetic acid [252] have been shown to influence pollen tube growth, indicating that these hormones can be perceived by the pollen tube and regulate its growth. Interestingly, IPs are also required for targeted pollen tube growth in Arabidopsis according to studies on *ipk2α* and *ipk2β* mutants [253]. The IPK2 enzymes catalyse the reactions from IP_3_ to IP_5_, successive phosphorylations of the D6- and D3-position of the inositol ring. Single mutants were phenotypically inconspicuous, however, a double mutant could not be obtained, likely due to a male transmission defect, which could be traced to impaired pollen germination and pollen tube guidance [253].

## 16. Diacylgylcerol Kinases Have Distinct Functions in Growing Pollen Tubes

In the animal field, DAG, the second product of PLC-activity, was established to act as a second messenger and to activate protein kinase C [254,255]. However, so far, no plant orthologues of these protein kinases have been identified [256]. Consequently, a role of DAG in plant cell signalling is under debate, especially since it can be easily phosphorylated by DAG kinases (DGKs) to form PA, which makes a dissection of DAG-specific and PA-specific reactions difficult [257,258]. So far, a DAG-specific role has only been proposed to act in the development of lateral root under mild salt stress [259]. Hence, the question whether DAG has a signalling role of its own is still unanswered. In pollen tubes, DAG localises to the apex and stretches through the subapical region towards the shank, overlapping with localisation of PI-PLCs, which seem to be primary responsible for its synthesis [239]. DAG localisation also overlaps with PA-localisation, as determined by a fluorescent lipid sensor. PA shows extended localisation in the pollen tube subapical flanks, however is not present at the tube apex [260]. While part of the PA originates from PLD enzyme activity, as discussed below, PA could also be produced from PI-PLC-derived DAG by a phosphorylation reaction (Figure 2). DGKs phosphorylate the free hydroxyl group at the *sn3*-position of the glycerol in DAG, yielding PA [261]. Inhibitor studies with the PLD-inhibitor 1-butanol and the DGK-inhibitor R59022 were carried out in tobacco pollen tubes as a first attempt to study the specific effect of DGK-produced PA [262]. Changes in vacuole morphology were thus traced to DGK activity, as vacuolar strands formed aggregates that extended into the tip region. In contrast, DGK-derived PA is apparently not involved in actin organisation during pollen tube growth [262]. Arabidopsis DGKs form a seven-membered protein family, of which *AtDGK4* is exclusively expressed in pollen, and proteomic analyses detected AtDGK4 and AtDGK5 in mature pollen grains. Similarly, a tobacco DAG kinase homologous to AtDGK5 was detected during all stages of tobacco pollen development [182,262,263]. Studies on one Arabidopsis T-DNA line of *AtDGK4* reported decreased pollen tube growth rates for *dgk4* pollen [264]. Pollen tubes of *dgk4* exhibited altered cell wall properties; furthermore, the authors reported impaired membrane recycling, and additional defects in the pollen tube reaction to NO have been reported [264,265]. However, another recent study did not report significant phenotypes in the single *dgk4* mutant, only for the double mutant *dgk2 dgk4* [266]. The respective enzymes DGK2 and DGK4 are localised to the ER and are probably involved in phospholipid metabolism there [266]. DGK function and DGK-derived PA have come into the focus of pollen tube research just recently, so the distinct sensors, transmitters, and effectors of PI-PLC/DGK-derived PA are still unexplored.

## 17. Phospholipase D-Produced Phosphatidic Acid Regulates Pollen Tube Growth

Unlike PLCs, which give rise to DAG, PLDs cleave phospholipids between the phosphate group and the headgroup, yielding PA [231,233]. In Arabidopsis, twelve PLDs are described differing in domain structure, enzymatic properties, and substrate preferences [243,267,268]. It is interesting to note that no Arabidopsis PLD activity on phosphoinositides has been reported, implying that PLC-mediated hydrolysis is the major pathway to terminate phosphoinositide signalling [243,268]. The origin of PLD-produced PA in pollen tubes is thus likely distinct from the PA produced by the concerted activity of PI-PLC and DGK, with the PI-PLC/DGK-pathway acting on phosphoinositides rather than structural phospholipids [235,269]. Nevertheless, PLD-activity has been demonstrated to be crucial for pollen tube growth using the PLD-inhibitor 1-butanol [270]. Subcellular analysis of tobacco pollen tubes treated with 1-butanol showed impairment of endocytosis, cell wall secretion, and actin regulation during tube growth [262]. The necessity of PA for exocytic processes has also been reported in various human cell types [271,272,273]. Two effects on secretion are generally discussed, firstly, that it could serve as a landmark in the plasma membrane, labelling the sites of exocytosis and recruiting proteins required for secretory processes. In this context, the anionic charge of its headgroup might help PA to interact with the positively charged domain of interacting proteins [272,274]. In line with this hypothesis, components of the tobacco exocyst complex partially colocalise with PA sensors in tobacco pollen tubes [215]. Secondly, PA influences biophysical properties of the membrane: its minimal headgroup results in a conical shape of the lipid, which in turn induces negative membrane curvature [274]. Membrane curvature is especially influential in vesicle formation so the shape of PA could be required for the endocytic recycling of vesicles at the pollen tube tip.

Several Arabidopsis PLD genes are expressed in pollen, and AtPLDα1, AtPLDα2, AtPLDβ1, and AtPLDδ are detected in mature pollen [182,262]. Despite this, no pollen tube growth phenotypes for Arabidopsis *pld* mutants have been described so far. In contrast, several tobacco PLDs are linked to pollen tube tip growth. Different *NtPLDs* were found to be expressed in pollen and germinated pollen, labelled NtPLDα2, NtPLDβ1, and NtPLDδ, and anti-sense-mediated knockdown of *NtPLDβ1* and *NtPLDδ* impaired pollen tube growth [275]. Furthermore, for NtPLDβ1, actin-binding capacity and regulation of PLD-activity by actin was demonstrated, leading to the conclusion that NtPLDβ1 is a key regulatory factor of actin organisation during pollen tube growth [275]. For NtPLDδ, a connection to ROS signalling was observed, as knockdown of NtPLDδ in tobacco pollen tubes led to a higher sensitivity towards H_2_O_2_. Interestingly, ROS-production by NtNOX itself is in part regulated by PLD-produced PA [62]. Recently, bioinformatic analysis of tobacco PLDδs classified five different NTPLDδs, NtPLDδ1 to 5 [276]. Overexpression of these PLDs in tobacco pollen tubes showed preferential cytosolic localisation for NtPLDδ1 and 2, plasma membrane localisation for NtPLDδ4 and 5, and an equilibrium between both compartments for NtPLDδ3. Tobacco pollen tubes overexpressing NtPLDδ3 showed the most severe phenotypes, including apical membrane invaginations. Due to the observed phenotypes, a role for NtPLDδ3 in membrane trafficking was proposed, highlighting the importance of lipid-mediated signalling for the maintenance of secretion in physiological tip growth [276].

## 18. Outlook

Many factors have been identified that are important for the polarisation of a pollen tube and its plasma membrane, including the channelling of secretory vesicles to the apical region by a highly specialised cytoskeleton and the regulation of exocytosis. This led to a model of a periodically oscillating system sustained and controlled by feedforward and feedback loops. In addition, external guidance cues and reacting receptors have been found. Now, the challenge emerges to understand how these external signals are integrated and transduced, so that cell wall properties are modulated that allow uneven tube extension and the pollen tube to “take a turn”. Interesting questions can also be asked about the beginning and the end of pollen tube growth: How is the cell polarity originally established and how is the cell wall weakening orchestrated that ultimately leads to the bursting of the pollen tube and release of the sperm cells at the correct place? Finally, we must understand how this fragile system adapts to environmental stress to sustain pollen tube growth and why it fails in some species but not in others. This could highlight strategies for the future to prevent crop losses under weather and climate extremes.
